# Genetic Diversity and Population Structure of Cowpea (*Vigna unguiculata* L. Walp)

**DOI:** 10.1371/journal.pone.0160941

**Published:** 2016-08-10

**Authors:** Haizheng Xiong, Ainong Shi, Beiquan Mou, Jun Qin, Dennis Motes, Weiguo Lu, Jianbing Ma, Yuejin Weng, Wei Yang, Dianxing Wu

**Affiliations:** 1 Department of Horticulture, University of Arkansas, Fayetteville, Arkansas, United States of America; 2 US Department of Agriculture, Agricultural Research Service (USDA-ARS), Salinas, California, United States of America; 3 Vegetable Research Center, University of Arkansas, Alma, Arkansas, United States of America; 4 State Key Lab of Rice Biology, IAEA Collaborating Center, Zhejiang University, Hangzhou, China; National Institute for Plant Genome Research, INDIA

## Abstract

The genetic diversity of cowpea was analyzed, and the population structure was estimated in a diverse set of 768 cultivated cowpea genotypes from the USDA GRIN cowpea collection, originally collected from 56 countries. Genotyping by sequencing was used to discover single nucleotide polymorphism (SNP) in cowpea and the identified SNP alleles were used to estimate the level of genetic diversity, population structure, and phylogenetic relationships. The aim of this study was to detect the gene pool structure of cowpea and to determine its relationship between different regions and countries. Based on the model-based ancestry analysis, the phylogenetic tree, and the principal component analysis, three well-differentiated genetic populations were postulated from 768 worldwide cowpea genotypes. According to the phylogenetic analyses between each individual, region, and country, we may trace the accession from off-original, back to the two candidate original areas (West and East of Africa) to predict the migration and domestication history during the cowpea dispersal and development. To our knowledge, this is the first report of the analysis of the genetic variation and relationship between globally cultivated cowpea genotypes. The results will help curators, researchers, and breeders to understand, utilize, conserve, and manage the collection for more efficient contribution to international cowpea research.

## Introduction

Cowpea (*Vigna unguiculata* L. Walp.), an annual crop, is one of the most important and widely cultivated legumes in the world, particularly in Africa, Latin America, and some parts of Asia and the United States. According to the data from the Food and Agriculture Organization (FAO) (http://www.fao.org), approximately 5.8 million tons of dry cowpea cereal is produced annually with a minimum of 11 million hectares planted all over the world. Cowpea cereal is a very important source of carbohydrates (63%) and proteins (25%), with low fat content (1.5%), and is rich in vitamins, minerals (Ca, P, Fe), folate, thiamin, and riboflavin. Cowpea is chiefly used as a grain crop; however, it also finds use as animal fodder or as a vegetable [1,2].

To breed new varieties with target traits, researchers need to have a better understanding of the raw breeding materials. An accurate selection of materials containing the desired gene may help to achieve the breeding objectives and shorten the breeding period. Outstanding cultivars are likely to be selected from a wider range of germplasm, on the other hand, from low-diversity germplasm, it is difficult to obtain high seed quality or new disease-resistant varieties [3]. Therefore, genetic diversity research is a crucial premise for breeding. From another perspective, long-term natural selection and artificial selection lead to certain genetic differences among crop populations and individuals. This is an important basis to cultivate new varieties of crops and to develop crop production. Along with the wide application of the molecular methods, breeding programs have remarkably expedited new cultivar release. Nevertheless, with the high-efficiency breeding process and new variety releases, some of the traditional local varieties have been gradually eliminated, resulting in narrowing of the genetic background of crop varieties. It is reported that in the last a few decades, Indonesia and China have lost 1,500 local rice varieties and 651 soybean varieties, respectively [4]. The shrinking genetic resources of crop varieties poses a great threat to agricultural production. Cowpea has a very long domestication history, and many Asian countries have ancient records of planting and cultivating cowpea 2000 years ago, which has resulted in cowpea facing a similar threat [3, 5, 6]. Therefore, protection, research, development, and utilization of plant genetic diversity are extremely important as the basis of cowpea breeding and genetic research. Genetic diversity research provides the basis of the genetic variation and genetic relationships among cowpea genotypes, thus providing information for the preservation and utilization of germplasm resources and improvement of cultivars [7].

The genetic diversity and relationship research of cowpea is a challenging topic for geneticists and breeders. In the last century the Vigna was initially divided and identified based on morphological traits [8]. However, morphological markers are easily influenced by the environment [9]. DNA markers such as restriction fragment length polymorphisms (RFLPs) [10], random-amplified polymorphic DNAs (RAPDs) [11–13], amplified fragment length polymorphisms (AFLPs) [6], inter simple sequence repeats (ISSRs) [14], and simple sequence repeats (SSRs) [15, 16] have been widely used for analysis of population structure and genetic diversity in plants. Single nucleotide polymorphism (SNP) has emerged as a powerful tool in genetic diversity studies as compared to other markers such as AFLP and SSR [17], because SNPs are abundant in the genomes of plants and other organisms [18]. Before 2012, to our knowledge, no cowpea diversity studies reported the use of SNPs markers [7]. In recent years, next generation sequencing (NGS) technologies using genotyping by sequencing (GBS)[19, 20] have been widely used for SNP discovery utilized in trait mapping associated with an inexpensive and fast approach[21, 22].

Previous cowpea genetic diversity researches revealed a clear but limited global picture because most investigators focused only on accessions from one or a few continents, and only a few researchers conducted genetic diversity studies using the entire global germplasm. Huynh et al [23] conducted genetic diversity and population structure analysis using SNP markers among 442 cowpea landraces collected throughout Africa and in other cowpea-growing regions of Asia, Europe, North America, and South America. The results revealed the presence of two major gene pools in cultivated cowpea in Africa. They described the relationship of cowpea landraces among the regions within and outside of Africa. However, only 15 samples collected from the American continent were included in their study. It would be beneficial to include more cowpea genotypes for genetic diversity analysis. Globally, it is difficult to find any reported current research on the worldwide genetic diversity of cultivated cowpea. Therefore, the diversity of cultivated cowpea from worldwide germplasm needs to be studied.

Genetic diversity research is often limited to domestication and phylogeny studies. There are several opinions on the origin and history of the ancient domesticated cowpea [24] and previous studies focused mainly on the local cowpea resource, especially in Africa and Asia [7]. West Africa and the Indian Sub-continent were considered the origin of cowpea domestication, according to earlier studies [25, 26]. With accumulation of evidence, the theory of Asian origin was unable to explain the traits and distribution of wild cowpea *Vigna dekindtiana* [27]. However, the intermedium-type of wild-domesticated cowpea, found in West and Central Africa, was considered proof of the West African origin center theory [8, 28, 29]. However, subsequent reports have not shown any consistent conclusions about the first domestication location for cowpea in Africa. Recently, 26 domesticated and 30 wild cowpea lines from West, East, and South Africa were analyzed by Ba et al. (2004) using RAPD markers. The results revealed wild species in East Africa having more polymorphisms, indicating that it may be the origin of cultivated cowpea [30]. Coulibaly et al. [31], using molecular markers, proposed that early domestication of cowpea occurred in Eastern Africa. Although whether Africa is the first domestication region remains uncertain, both West and East domestication theories are now widely accepted. Nonetheless, compared to many other important crops, cowpea is relatively little understood with respect to the relationship of dispersal and development among first domestication region, sub-domestication region, and cultivated regions.

A diverse set of 768 cultivated cowpea genotypes, distributed in 58 countries, was included in this study. Besides the cowpea accessions from Africa, several accessions from Asia, America, and Oceania continents were also included in this study to analyze the genetic diversity among all geographic regions and the relationship among geographic regions. GBS was used to discover SNP in the cowpea set, and SNPs postulated from GBS were used to estimate the level of genetic diversity, population structure, and phylogenetic relationships. The study aimed to detect the gene pool structure of cowpea and to determine its relationship among different regions. According to the phylogenetic relationship between each individual, region and country, we attempted to trace the accession from off-original regions back to the two candidate original areas (West and East of Africa) to predict the migration and domestication history during the cowpea dispersal and development. The objective of this study was to systematically analyze the genetic variations and relationship among globally cultivated cowpea genotypes and to conduct population structure analysis of the species in order to put forth information for curators, researchers, breeders to utilize, conserve, and manage cowpea germplasm accessions and cultivars in cowpea breeding and other research programs.

## Materials and Methods

### Plant material

A total of 768 cowpea genotypes, collected from 56 countries, were used in this study. Among them, 716 accessions were obtained from the US National Plant Germplasm System (NPGS, http://www.ars-grin.gov/npgs/), and 52 local cultivars and breeding lines were taken from the University of Arkansas breeding program. The accessions were mainly from four regions consisting of 567 lines, India having 160 accessions, North America 162 (including 74 American cultivars), South Africa 133, and West Africa 112; and the other regions had 201 cowpea lines: Central East Africa 25, East Asia 26, Europe 8, Oceania 9, Central West Asia 66, and Latin America 67 (Table 1, S1 Table).

**Table 1:** Allelic analysis of 768 cowpea accessions from 11 geographic regions and genotyped with 1,048 SNP markers.

Region	No. accessions	Major Allele Frequency (%)	No. Countries	Gene Diversity	Heterozygosity	PIC	Country
**American Cultivars**	74	77	1	0.27	0.05	0.23	US
**North America**	88	76	1	0.3	0.05	0.25	US
**Latin America**	67	77	17	0.3	0.06	0.26	Argentina, Brazil, Colombia, Costa Rica, Cuba, El Salvador, Guatemala, Honduras, Les Cayes, Mexica, Nicaragus, Paraguay, Peru, Puerto Rico, Suriname, Trinidadand Tobago, Uruguay
**Europe**	8	85	2	0.21	0.06	0.17	Hungary, Portugal
**East Asia**	26	79	9	0.28	0.06	0.23	China, Former Soviet Union, Indonesia, Japan, Myanmar, Philippines, Sri Lanka, Taiwan, Thailand
**Central West Asia**	66	80	6	0.28	0.05	0.23	Afghanistan, Iran, Israel, Lebanon, Pakistan, Turkey
**Oceania**	9	78	1	0.32	0.05	0.28	Australia
**India**	160	72	1	0.35	0.08	0.32	India
**Central East Africa**	25	77	7	0.34	0.05	0.3	Congo, Egypt, Ethiopia, Kenya, Tanzania, Uganda, Zaire
**South Africa**	133	73	7	0.33	0.06	0.28	Botswana, Malawi, Mozambique, South Africa, Zambia, Zimbabwe
**West Africa**	112	72	7	0.22	0.06	0.27	Burkina Faso, Burundi, Cameroon, Ghana, Niger, Nigeria, Senegal

### DNA extraction, sequencing, and SNP calling

Five seeds from each cowpea line were planted in pots in the greenhouse. Two- weeks- old leaves from each accession were bulked into a single sample. The genome DNA was isolated from approximately one gram of leaf tissue from each bulk using a CTAB-based method [32]. The DNA quality and concentrations were detected by electrophoresis gel and a Nano Drop 2000 Spectrophotometer (Thermo Scientific, Wilmington, DE, USA). The DNA samples with a bright band and a concentration of more than 400 ng μl^-1^ were selected and transported to the Beijing Genome Institute (BGI) for GBS [19, 20] and SNP calling. DNA normalization, library preparation, and GBS were conducted by HiSeq 2000 in the BGI. The raw sequencing data and SNP calling were analyzed by BGI using SOAP family software (http://soap.genomics.org.cn/). The SOAPaligner/soap2 (http://soap.genomics.org.cn/) was used to align the short-read to cowpea genome reference (cowpea_Genome_0.03.fa) and SOAPsnp v 1.05 was used for SNP calling [33, 34]. The cowpea_Genome_0.03.fa (6,750 scaffolds or contigs) (http://harvest-blast.org/) was kindly provided by Dr. Timothy J. Close at the University of California, Riverside, U.S.A.

### Population structure

The model-based program STRUCTURE 2.3.1 [35] (http://pritchardlab.stanford.edu/software/structure_v.2.3.1.html) was used to infer the population structure. In order to identify the number of populations (K) the capturing of the major structure in the data, we set up at a burn-in period of 10,000 Markov Chain Monte Carlo iterations and 100,000 run length, with an admixture model following Hardy-Weinberg equilibrium and correlated allele frequencies as well as independent loci for each run [35]. Ten independent runs were performed for each simulated value of K, ranging from 1 to 11. Subsequently, the optimal K was determined using Structure Harvester [36] (http://taylor0.biology.ucla.edu/structureHarvester/).

### Genetic diversity

For each SNP, the major allele frequency, heterozygosity, gene diversity, and polymorphism information content (PIC) were calculated using the PowerMarker V3.25 software, and the genetic diversity for the entire set of cowpea genotypes as well as the geographically based sub-populations were also identified by PowerMarker version 3.25 [37], using genetic distances with CS Chord 1967 method [38]. Analysis of molecular variance was performed with the software Arlequin 3.5 [39] applied to all informative markers. Phylogenetic relationships and principal component analysis (PCA) were generated by TASSEL 5.2.13 to analyze genetic relationships among accessions and to determine the optimal number of clusters in the study. Phylogenetic Tree based on the genetic-distance among regions or countries was calculated by using neighbor-joining method [40] in the function of PowerMarker version 3.25 and was visualized by software MEGA 6 [41].

## Results

### Single nucleotide polymorphism diversity

Totally 5,828 polymorphic SNPs with less than 50% missing across 768 accessions/cultivars were obtained from the BGI. These were subsequently filtered by removing the rare alleles (less than 5%), high-missing ratios (more than 30%) and the high heterozygosity alleles (more than 70%). The finally selected 1,048 SNPs were subjected to genetic analyses and six SNP types were determined from them as follows: [AG] SNP type had 270 SNPs (25.7%); [CT] 246 (23.5%); [GT] 149 (14.2%); [AT] 139 (13.3%); [AC] 127 (12.1%); and [CG] 118 (11.2%). Among the 1,048 SNP loci, the major allele frequency, gene diversity, heterozygosity, and PIC averaged 0.77, 0.32, 0.06 and 0.26, respectively, and also showed large ranges of; 0.50–0.95, 0.09–0.52, 0.0–0.68, and 0.08–0.41, respectively, indicating the existence of SNP variations and mutations (gene flow) and also genetic diversity among the 768 cowpea genotypes.

### Population structure and genetic diversity

**Fig 1:**
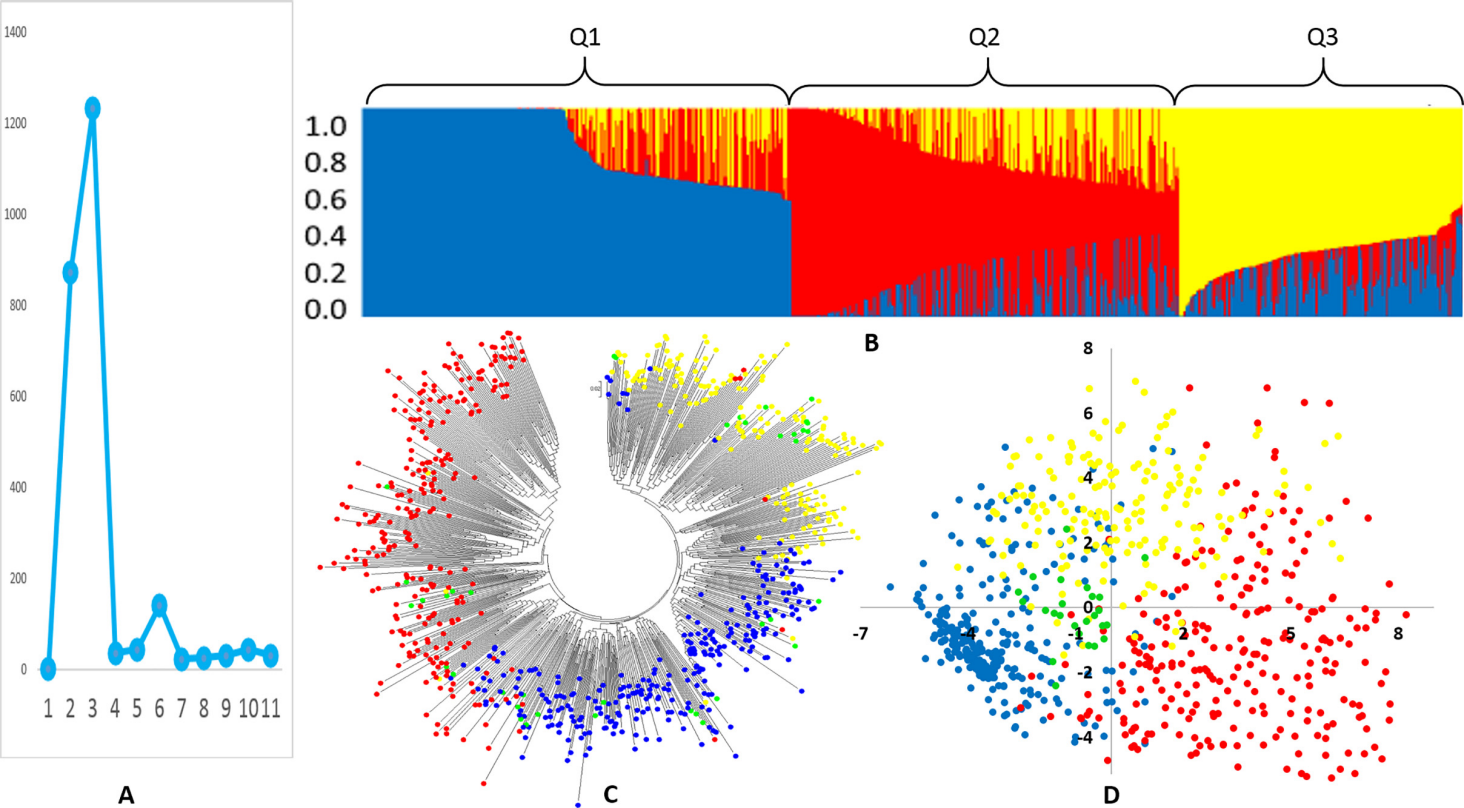
(A) Delta K values for different numbers of populations assumed (K) in the STRUCTURE analysis. (B) Classification of 768 accessions into three populations using STRUCTURE 2.3.1. The distribution of the accessions to different populations is indicated by the color code. Numbers on the y-axis show the subgroup membership, and the x-axis shows the different accession.(C)Unrooted Neighbor-Joining (NJ) tree of the 768 accessions drawn by MEGA 6 and each colored shape represents one cluster matching the structure population (blue for Q1, red for Q2, yellow for Q3, and green for admixture).(D)the scatter diagram of Principal Component Analysis (PCA) of the 768 accessions, calculated by TASSEL and drawn by Excel and each colored spot is representative of one cluster Q1 to Q3 same as in (C).

The population structure of the 768 cowpea accessions/cultivars was inferred using STRUCTURE 2.3.4 [42] and the peak of delta K was observed at K = 3, suggesting the presence of three main populations (clusters, Q1, Q2, and Q3) in the cowpea panel (Fig 1A and 1B). The classification of accessions into populations based on the model-based structure from STRUCTURE 2.3.4 is shown in Fig 1B and the S1 Table. Using 0.55 as the likelihood to cluster for each accession in the three populations, a total of 732 accessions/cultivars (95.2%) were grouped to one of the three populations. The first cluster of 288 (37.5% of total accessions/cultivars) accessions was grouped into Q1, the next 260 (33.9%) into Q2, and 183 (23.8%) into Q3 (Fig 1B, S1 Table). The remaining 37 out of the 768 accessions (4.8%) were placed in the admixture (S1 Table).

Neighbor-joined cluster analysis from MEGA 6 [41] also clearly divided the 768 accessions into three groups (Fig 1C), which was consistent with the model-based population structure from STRUCTURE. The distribution of the two dimensions created by principal component analysis (PCA) on all 768 accessions (Fig 1D) also supported the separation of the accessions into three clusters, which was also consistent with the model-based population structure. In summary, the model-based ancestry analysis, the phylogenetic tree and the PCA strongly supported that cowpea had three well-differentiated genetic populations and admixtures.

### Genetic diversity by region

In this study, the tested 768 cowpea accessions, except the Indian accessions, were divided into 9 groups based on their original geographical regions: South Africa, West Africa, Central East Africa, East Asia, Central West Asia, Europe, Oceania, North America, and Latin America; the germplasm accessions from India was listed as a special group called “India” because West Africa and the Indian Sub-continent were considered to be the origin of cowpea domestication according the earlier studies [25, 26]; and the cultivars from US was also placed in a separate group named “American Cultivars” because they were more developed as cultivars for cultivation by farmers (Table 1, S1 Table).

Based on the 11 groups, the genetic parameters of each of the 11 groups were estimated for the number of cowpea accessions, number of countries, the major allele frequency, gene diversity, heterozygosity, and PIC (Table 1). Nine of the groups had 25 or more cowpea accessions however; Europe and Oceania had only 8 and 9 accessions, respectively. The countries in each group were also listed in Table 1, where the accessions in “North America” were all collected from the U.S.A; the “Oceania” group from Australia; the special groups “India” and “America Cultivar” from India and the U.S.A., respectively. The gene diversity varied in different groups from 0.21 in Europe to 0.35 in India, indicating genetic variation in each group. The heterozygosity was 0.05 or 0.06, representing that there was 5 or 6% heterozygosity of alleles existing in each group and most of the alleles in the cowpea accessions of each group were fixed with homozygosity. The PIC ranged from 0.17 in Europe to 0.32 in India, which was similar to the gene diversity, indicating that the accessions in Europe had the least variation and the accessions in India varied the most.

**Fig 2:**
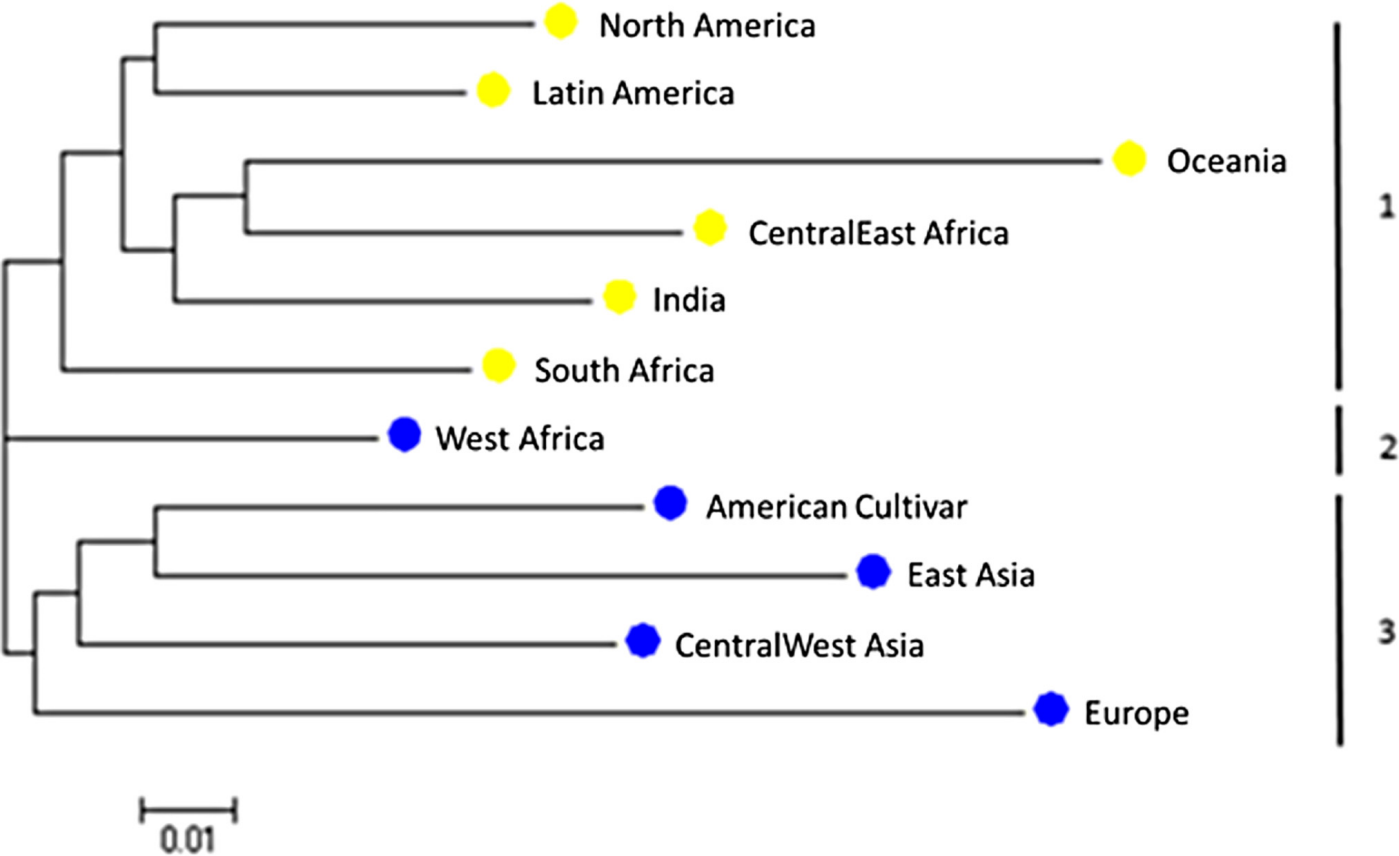
Phylogenetic tree among 11 regions based on the genetic-distance matrix using the neighbor-joining method by Power Marker V3.25 and visualized using the software MEGA 6. The blue and yellow balls in each region represent the accession ratio of cluster Q3 ratio in region: blue for low (less than 10%) and red for high (greater than 20%) (Table 2).

The genetic distance among the 11 groups was calculated using CS Chord 1967 [38] method in PowerMarker version 3.25 [37]. The phylogenetic tree drawn using neighbor-joining method [40] was visualized by software MEGA 6 [41] (Fig 2), and divided into three clusters where, North America, Latin America, Oceania, Central East Africa, India, and South Africa together formed the cluster 1; West Africa alone comprised the cluster 2; and the American Cultivar, East Asia, Central West Asia, and Europe were placed in the cluster 3 (Fig 2). The cowpea genotypes in the same cluster displayed closer genetic backgrounds.

The population structure in each of the 11 groups was further analyzed based on the results from STRUCTURE 2.3.1 (Fig 2). Three clusters (Q1, Q2, and Q3) were observed in the all tested 768 cowpea genotypes (Fig 1). However, the numbers and the percentage of accessions in each of the 11 groups were different (Table 2). Eight of the 11 groups except East Asia, Europe, and Oceania had three structured populations (clusters Q1 to Q3); East Asia had two clusters Q1 and Q2 but lacked Q3; Europe exhibited only one cluster (Q1); and Oceania had Q2 and Q3 but no Q1. In cluster 1 (Q1), the majority of accessions (above 10%) came from West Africa (51 accessions; 17.7%), India (49 accessions; 17.0%), North America (42 accessions; 14.6%), Latin America (30 accessions with 10.4%), and American Cultivars (35 accessions; 12.2%), representing 71.9% of the total of Q1 accessions. In cluster 2 (Q2), the majority of accessions came from South Africa (58 accessions; 22.3%), West Africa (51 accessions; 19.6%), Central West Asia (34 accessions; 13.1%), and American Cultivars (34 accessions; 13.1%), representing 68.1% of the total of Q2 accessions. In cluster 3 (Q3), the majority of accessions came from India (80 accessions; 43.7%), South Africa (36 accessions; 19.7%), and North American (19 accessions; 10.4%), representing 73.8% of the total of Q3 accessions. The different accession numbers and percentage of each cluster among the 11 groups and the different accession percentage of each group among the three clusters revealed that a geographical or a regional factor existed for cowpea genetic diversity and population structure.

**Table 2:** Number and percentage of cowpea accessions in clusters among the 11 regions.

Region	No. of accessions in each cluster				Percentage of accessions in each cluster				Total No. of accessions in each region
	Q1	Q2	Q3	admixture	Q1	Q2	Q3	admixture	
American Cultivars	35	34	3	2	47.3	45.9	4.1	2.7	74
North America	42	20	19	7	47.7	22.7	21.6	8.0	88
Latin America	30	18	17	2	44.8	26.9	25.4	3.0	67
Europe	8	0	0	0	100	0	0	0	8
East Asia	8	17	0	1	30.8	65.4	0	3.8	26
Central West Asia	25	34	6	1	37.9	51.5	9.1	1.5	66
Oceania	0	1	8	0	0	11.1	88.9	0	9
India	49	20	80	11	30.6	12.5	50.0	6.9	160
Central East Africa	12	7	6	0	48.0	28.0	24.0	0	25
South Africa	28	58	36	11	21.1	43.6	27.1	8.3	133
West Africa	51	51	8	2	45.5	45.5	7.1	1.8	112

For each of the 11 groups, the majority of accessions were sectored into different structured populations (clusters). For the two groups; America Cultivar and West Africa, the majority of accessions were divided into two populations (cluster Q1 and Q2) with nearly half each; in the other three groups: North America, Latin America, and Central East Africa, the majority of accessions belonged to Q1 with nearly 50% accessions, and the other half were distributed in Q2 and Q3; Europe had 100% accessions in Q1; for the East Asia, Central West Asia, and South Africa group, nearly half to more than half were in Q2; a total of half the accessions in India were located in Q3 (50.0%), and 88.9% accessions in Oceania were in Q3 (Table 2), further emphasizing that geographical factors play a role in the cowpea genetic diversity and population structure.

Based on the percentage of accessions in the structured population 3 (Q3) in each of the 11 groups, two larger groups can be constructed: i) a high Q3 ratio group including North America (21.6%), Latin America (25.4%), Oceania (88.9%), India (50.0%), Central East Africa (24.0%), and South Africa (27.1%), and ii) a low Q3 ratio group consisting of America Cultivar (4.1%), Europe (0%), East Asia (0%), Central west Asia (9.1%), and West Africa (7.1%), which is similar to the phylogenetic relationship in Fig 2 created by PowerMarker drawn by MEGA 6.

### Genetic diversity by country

**Fig 3:**
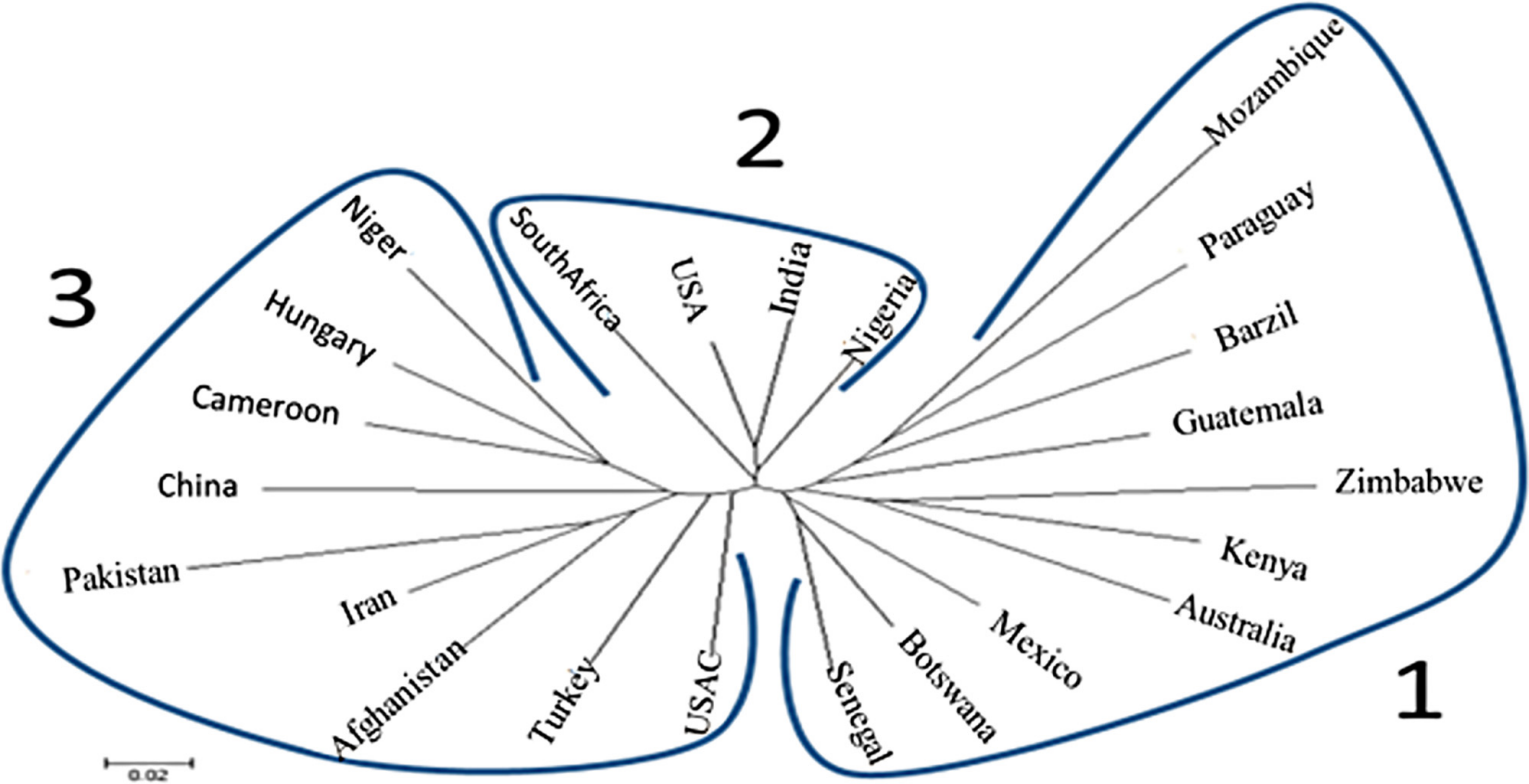
Phylogenetic diagram among countries based on the genetic-distance matrix using the neighbor-joining method by Power Marker V3.25 and visualized using the software MEGA 6. Three Clusters were divided among 22 countries plus the US cultivars (USC) (Table 3).

The genetic diversity was further analyzed by country in all 768 tested cowpea genotypes originally collected from 56 countries with 22 out of the 56 countries showing 5 or more cowpea accessions (S1 Table). A sub-total of 705 accessions from the 22 countries was further studied for genetic diversity based on its country of origin. The genetic distances among the 22 countries were obtained with CS Chord 1967 method [38] by PowerMarker [37] and the phylogenetic tree was created and viewed using MEGA 6 (Fig 3). The results obtained were differentiated into three distinctive clusters. Nine countries were placed in Cluster 1, where five were from Asia (Afghanistan, Iran, Pakistan, Turkey, and China), two were from West Africa (Cameroon and Niger), and one was from Europe (Hungary) plus the America Cultivar; Cluster 2 consisted of four countries: Nigeria, South Africa, India, and US; and Cluster 3 included ten countries, four from Latin America (Brazil, Guatemala, Mexico, and Paraguay), three from South Africa (Botswana, Mozambique, and Zimbabwe), and one each from West Africa (Senegal), Central East Africa (Kenya) and Oceania (Australia) (Table 3). The three clusters created by country were similar to those by region (Table 3). Eighteen of the 24 countries with the exception South Africa, India, US, Senegal, and f two from West Africa (Cameroon and Niger), were divided into the corresponding three clusters (Table 3), indicating a similarity of results from two genetically diverse approaches.

**Table 3:** Cluster analysis by country including 22 countries with 5 or more cowpea accessions.

Country	No. of accessions	Cluster by Country	Region	Cluster by region
USC	74	1	America Cultivar	1
Afghanistan	16	1	Central West Asia	1
Iran	12	1	Central West Asia	1
Pakistan	5	1	Central West Asia	1
Turkey	31	1	Central West Asia	1
China	15	1	East Asia	1
Hungary	5	1	Europe	1
Cameroon	17	1	West Africa	2
Niger	9	1	West Africa	2
Nigeria	47	2	West Africa	2
South Africa	25	2	South Africa	3
India	160	2	India	3
U.S.A.	88	2	North America	3
Senegal	29	3	West Africa	2
Botswana	96	3	South Africa	3
Mozambique	5	3	South Africa	3
Zimbabwe	5	3	South Africa	3
Kenya	13	3	Central East Africa	3
Australia	9	3	Oceania	3
Brazil	7	3	Latin America	3
Guatemala	9	3	Latin America	3
Mexico	20	3	Latin America	3
Paraguay	8	3	Latin America	3

## Discussion

The genotyping by sequencing (GBS) using the Illumina HiSeq showed high levels of SNP variations among the cowpea samples. The percentage of each SNP type in our study was 25.7, 23.5, 14.2, 13.3, 12.1, and 11.2% for [AG], [CT], [GT], [AT], [AC], and [CG], respectively, which complied with previously reported research [23] where 1536-SNPs of the Golden Gate genotyping assay was used. Our research confirmed that the SNP variations existed in cowpea germplasm and the [AG] or [CT] SNP types were more prevalent in cowpea than other types.

The majority of genetic variance exists within instead of among geographic regions and within instead of among countries in the USDA cowpea world collection. Compared to the study by Zannouou et al. [43] with accessions from Benin, the genetic variances among populations (regions and countries) from this study were very low. However, there are some reports with similar results in the accessions from Sudan [44] and Ghana [45] [46]. Several other studies like the one on Phleum, also did not observe significant correlation between the various accessions and their geographic origins [47]. The explanation for this phenomenon might be because of the different germplasm used.

Population structure analyses divided the 768 cultivated cowpea genotypes into 3 gene pools (clusters Q1, Q2, and Q3) based on the peak of delta K (DK) at K = 3. While the DK had a high score at K = 2 (Fig 1 B), in accordance with previous studies showing that the landrace germplasm was divided into two clusters as two gene pools which were distributed in two geographical regions of Africa. The landraces from gene pool 1 were mostly distributed in western Africa while the majority of gene pool 2 were located in eastern Africa [23]. However, observing the results of structure analysis, the two gene pool system perhaps was not the best choice in our study. Based on the population structure analysis by STRUCTURE (Fig 1) the three gene pool system was the best fit and also delivered better results based on the geography of region and the country using genetic distance analysis by PowerMarker viewed on MEGA to create phylogenetic trees (Figs 2 and 3). The three gene pools (3 clusters) was efficient in distinguishing the populations within the three regions of America (American Cultivar, North America, and Latin America), three African regions (Central East Africa, South Africa, and West Africa), Central West Asia, and India except East Asia, Europe and Oceania (Table 2). The distinctions between populations within limited local areas corroborated with prior reports [3, 23, 26]. Although both the two gene pool- and three gene pool-systems cannot segregate the populations among each continent completely, the three gene pool system had a better performance within the local area. The obscure separation between each continent may be caused by 1) material exchange and transfer during cowpea breeding improvement, 2) the multiple usage of cowpea. These two reasons can also explain the phenomenon that the majority of genetic variance exists within instead of among geographic regions in world cowpea collection [3, 26]. Multiple types of cowpea are often planted in the same continent for vegetable, grain, and fodder. This planting habit would dramatically increase the diversity in the local area and decrease the genetic distance among continents. In addition, the distinctions between each region in same continent might also be caused by cowpea usage based on local dietary habit.

Based on PIC values, the accessions in the cowpea collection that originated in India and East Africa are most highly diversified (3.2 and 3.0), followed by Oceania, America, South and West Africa with medium high diversity, then the American Cultivar, East and Central West Asia with medium and Europe with lowest PIC (0.17). Different growing environments, availability of genetic stocks, and diverse cowpea consumption behaviors may be responsible for diversity differences among the regions. In our study the diversity degree compared to prior landrace study have been slightly reduced [23], which may be due to long-term cultivation or breeding.

In general, the degree of genetic diversity tends to have a positive correlation with the number of countries from which the accessions were collected. The more the number of origins of accessions, the more is the genetic diversity detected [4]. Truly, this regulation would explain the high genetic diversity of accessions from South and West Africa, which contain 7 and 8 countries, respectively. It also can explain the lowest genetic diversity of accessions from Europe with only 2 countries. Nevertheless, we still observed exceptions like: India, North America and Oceania, which contains one country each but had high PIC (0.32, 0.25 and 0.28), which might be explained by the higher number of cowpea accessions from India (160) and North America (88) but not for Oceania with only 9 accessions. The accessions from East Asia (9) and Latin America (17) did not get the expected high PIC. In our study, the number of origins may not be the factor to influence the PIC and the genetic distances among cowpea genotypes are low due to the inherent self-pollination mechanism of the cowpea [46]. In our study the genetic distance, genetic structure and genetic diversity analysis had a high consistency and accordance. The populations from different areas with similar genetic structure always have a smaller distance and similar genetic diversity and vice versa. We can observe that the populations from 6 regions (Table 2) with high Q3 ratio, India, North America, Latin America, Oceania, South and East Africa, not only have high PIC (Table 1) but also are grouped together in Cluster 1 (Fig 2).The same phenomenon was also observed in another cluster with 4 regions (American Cultivars, East and Central West Asia and Europe) with low Q3 ratio. The only exception was the population in West Africa that had high PIC and low Q3 ratio and was related closely to Cluster 1 (Q1) and Cluster 3 (Q3).

In the African continent, the populations belonging to each region have very low genetic distance, but the populations between West and South, and the Central East have an obvious difference in Q3 accessions ratio. One possibility for this paradox is that the Q3 accessions have high homology with Q1. This is also authenticated by the phylogenetic result (Fig 1C) which showed that the Q1 and Q3 clusters on a same main branch and the PCA result (Fig 1D) which revealed more overlap between Q1 and Q3. Given the context, the plausible hypothesis is that the Q3 gene pool may be derived from the Q1 gene pools by any of the possible reasons like hybridization, gene flow or cultivar localization during the domestication. This hypothesis would explain why the populations in India has the most Q3 and PIC accessions in our study. We also found a very short genetic distance between India and Central East Africa, which implies the movement via human migration from Central East Africa to India that is known as sub-domestication region. This import and domestication occurred for a long time. Cowpea grown in such vast areas in the regions must adapt to complex environmental conditions in terms of temperatures, water availability, elevations, soil types which maybe the reason why India was recognized as the secondary center of cowpea diversity [25]. The most special forms of cowpea are *sesquipedalis* and *biflora*, which can only be found in India and East Asia. These two types differ from the African domesticated forms. The possible assumption is that when cowpea moved farther east into East Asia and encountered more humid environments with less sunshine, unsuitable for drying pods and grains it made people prefer the immature pods as vegetable in Asia [23]. During this domestication and import of cowpea in East Asia, the two types of cowpea were formed and adopted to local diet habits especially in China and the Southeast Asian countries [48]. The cluster of India, Oceania, North and Latin America, also suggests that the cowpea in the America continent and Oceania may have come from India during the colonization of the British.

The wield distribution of countries from West Africa was found in the Country Cluster Analysis (Fig 3), which can describe the relationship and correlation among regions. This result can well explain why West Africa barely clusters into group 1 or 2 (Fig 3). The accessions from Europe cluster with the two West Africa countries in the same branch, which implies that Europe might have imported the cowpea directly from West Africa.

The closest relation was found between North and Latin America, also having a highly similar genetic structure and PIC, which reveals a high accordance in variety import and localization. In addition, low Q3 ratio in America Cultivar implies that breeding programs in America might import and employ a large number of the accessions from West Africa or Asia. A similar report was also brought out by Fang et al [6] who indicated that the America breeding lines have minimum 86% similarity with the accessions from West Africa. The non-Cultivar accessions in America are not close to those from West Africa, which was also reported by Huynh et al [23] who compared landraces between the two regions. That may be the reason behind the differences in PIC and genetic structure in the American non-cultivar and cultivar. The difference between cultivar and non-cultivar accessions in genetic structure and distance indicated that there are still abundant genetic resources in breeding materials, which show good adaptation in local areas, especially under biotic and abiotic stress environments. The accessions from India were more diverse than the accessions from East Asia, Oceania, North and Latin America, which may suggests a bi-direction of bottlenecks or founder effects during cowpea domestication and diffusion.

Breeding projects could generally narrow the genetic variation of crop resources. If there were no germplasm introduced into the programs, genetic diversity would be reduced over time. In our study, we came to the same conclusion that breeding dramatically reduces the genetic variations of cowpea. We also found that some new cowpea resources were created when it was moved or domesticated into new environments, for example, in East Asia, which may increase the genetic diversity of the breeding groups. Now geographical barrier has now drastically reduced when the germplasm involved into whole cultivation system in the world during the globalization, which is the chance to help us improve the breeding resources. We have to consider both phenotype and genotype aspects in understanding germplasm resources, especially in breeding programs.

## Conclusion

Three well-differentiated genetic populations called structured populations or clusters were postulated from this study in the 768 world-wide cowpea genotypes based on genome-wide SNPs. The populations (clusters) were associated with the regions and countries where the cowpea genotypes were collected. Cluster 1 mainly consisted of the cowpea genotypes from Asia (Afghanistan, Iran, Pakistan, Turkey, and China), West Africa (Cameroon and Niger), and Europe (Hungary) plus the American Cultivars; Cluster 2 was composed of accessions from South Africa, India, and US; and Cluster 3 was from Latin America (Brazil, Guatemala, Mexico, and Paraguay), South Africa (Botswana, Mozambique, and Zimbabwe), Central East Africa (Kenya) and Oceania (Australia). This study supports the two candidate theory of the original areas (West and East of Africa) as the first domestication regions of cowpea and India as a sub- domestication region of cultivated cowpea.

## Supporting Information

S1 Table Cowpea accession, name, taxon, region, country, location collected, and cluster assigned in this study. (XLSX)
